# Interventions addressing functional abilities of older people in rural and remote areas: a scoping review of available evidence based on WHO functional ability domains

**DOI:** 10.1186/s12877-022-03460-2

**Published:** 2022-10-28

**Authors:** Ivy Yan Zhao, Jed Montayre, Angela Y. M. Leung, Jann Foster, Ariana Kong, Stephen Neville, Ramona Ludolph, Christopher Mikton, Alana Officer, Alex Molassiotis

**Affiliations:** 1 WHO Collaborating Centre for Community Health Services, School of Nursing, The Hong Kong Polytechnic University, SAR Hung Hom, Hong Kong; 2 New South Wales Centre for Evidence-Based Healthcare - JBI affiliated group, School of Nursing and Midwifery, Western Sydney University, 2751 Penrith, NSW Australia; 3 South Western Sydney Local Health District, Ingham Institute for Applied Medical Research, 2170 Liverpool, NSW Australia; 4 School of Nursing and Midwifery, Western Sydney University, Locked Bag 1797, 2751 Penrith, NSW Australia; 5 Centre for Oral Health Outcomes and Research Translation (COHORT), School of Nursing and Midwifery, Western Sydney University, 2751 Penrith, NSW Australia; 6 School of Clinical Sciences, Auckland University of Technology, 90 Akoranga Drive, Northcote, Auckland, New Zealand; 7 World Health Organization, Geneva 27, 1211 Geneva, Switzerland; 8 Health & Social Care Research Centre, University of Derby, Derby, United Kingdom

**Keywords:** Healthy ageing, Functional ability, Interventions, Rural and remote, Ageing, Scoping review

## Abstract

**Background:**

The World Health Organization (WHO) encourages healthy ageing strategies to help develop and maintain older people’s functional abilities in five domains: their ability to meet basic needs; learn, grow, and make decisions; be mobile; build and maintain relationships, and contribute to society. This scoping review reports the available evidence-based interventions that have been undertaken with people ≥ 50 years of age in rural and remote areas and the outcomes of those interventions relevant to enhancing functional ability.

**Methods:**

The scoping review was undertaken following the JBI methodology. A literature search was carried out to identify published intervention studies for enhancing functional ability in older people living in rural and remote settings. The databases searched included CINAHL, Scopus, ProQuest Central, PubMed, EBSCOHost, APA PsycInfo, Carin.info, and the European Network for Rural Development Projects and Practice database. Gray literature sources included government reports, websites, policy papers, online newsletters, and studies from a bibliographic hand search of included studies.

**Results:**

Literature published from January 2010 to March 9, 2021 were included for review. A total of 67 studies were identified, including quasi-experimental studies (n = 44), randomized controlled trials (n = 22), and a descriptive study. Five main types of interventions were conducted in rural and remote areas with older people: Community Services, Education and Training, Exercise and Physical Activity, Health Promotion Programmes, and Telehealth. Health Promotion Programmes (n = 28, 41.8%) were the most frequently reported interventions. These focused primarily on improving the ability to meet basic needs. About half (n = 35, 52.2%) of the included studies were linked to the ability to learn, grow, and make decisions, and 40% of studies (n = 27) were relevant to the ability to be mobile. Only a very limited number of intervention studies were geared towards outcomes such as maintaining relationships (n = 6) and contributing to society (n = 3).

**Conclusion:**

Interventions for enhancing functional ability focused primarily on the ability to meet basic needs. We identified the need for health-related interventions in rural and remote areas to consider all five functional ability domains as outcomes, particularly to strengthen the psychosocial wellbeing of older people and enhance their sense of purpose through their contributions to society.

**Supplementary Information:**

The online version contains supplementary material available at 10.1186/s12877-022-03460-2.

## Introduction

By 2050, one in six people will be 65 years of age or over [1]. Demographic ageing in rural and remote areas reflects similar trends to the ageing of the global population [2]. Although a significant demographic shift has taken place in the past decades in relation to rural-to-urban migration, an increasing number of older people are residing permanently in rural and remote areas [3]. Living in such places comes with some common and unique challenges to ageing residents. Rural and remote areas have lower population densities and inhabited areas are geographically dispersed, making it more difficult to fund and create comprehensive services such as health and social infrastructure for small numbers of older people scattered over large areas [4].

Current research on rural and remote communities shows that older people face unique social and environmental challenges. For example, Europe’s growing rural population of older people are at greater risk of social isolation or loneliness than those in urban areas, are placing increased demands on health and social care services, and are encountering significant difficulties in finding service providers [5]. In Canada, an estimated 23% of older adults live in rural communities and are facing the specific challenges of restricted access to certain health and social services, as well as limited housing and transportation options [6]. In many Asian societies, the emphasis on filial piety is more often observed in rural communities. However, with the migration of younger generations to cities for employment and the resulting lack of family support, the mental health of older parents can be negatively impacted by financial issues and loneliness [6]. A review of life expectancies and disability-free expectancies since 2010 in the UK found that people living in areas lacking infrastructure and resources may have shorter lives due to these limitations [7]. Consequently, these challenges have put older populations in rural and remote areas at a considerable disadvantage compared to urban residents, which negatively impacts their health and longevity.

The World Report on Ageing and Health [8] defines healthy ageing as ‘the process of developing and maintaining functional ability that enables wellbeing in older age’. According to this definition [8], functional ability consists of the intrinsic capacity of the individual, relevant environmental characteristics, and the interaction between them. The WHO encourages strategies to help develop and maintain older people’s functional abilities in five domains: to (a) meet their basic needs, (b) learn, grow, and make decisions, (c) be mobile, (d) build and maintain relationships, and (e) contribute to society [9]. The WHO’s International Classification of Functioning, Disability and Health (ICF) framework is designed for organizing and documenting information on functioning and disability [10]. Functioning is conceptualized as a ‘dynamic interaction between a person’s health condition, environmental factors and personal factors’ and disability is denoted as a ‘negative outcome or result of a complex relationship between an individual’s health condition and personal factors, and of circumstances in which the individual lives’ [10].

Most existing studies on functional ability interventions and functional assessment methods have focused mainly on a particular type of disease or on the performance of activities of daily living and/or on the mental capacity of older people [11]. For example, the most commonly used standards for assessing functional ability in community-dwelling older adults were the Activities of Daily Living (ADL) Scale [12], the gait speed test, the chair stand test, and handgrip-strength test [13–15], and the Geriatric Depression Scale (GDS) [16]. However, research on activities relating to socialization and communication are often limited, despite the relationship between social engagement and functional decline [11]. Moreover, little is known about interventions that foster functional ability in older people who live in remote and rural areas.

## Review aim

The overall aim of this scoping review was to identify and examine evidence-based intervention studies implemented in rural and remote areas with older adults as the target population. The review sought to answer the following specific questions:

1. What interventions have been implemented in rural and remote areas that contribute to enhancing the five domains of functional ability for older people?
2. What were the most common evaluated outcomes from these interventions in relation to developing and maintaining the five domains of functional ability?

## Methods

The current review was undertaken following the JBI methodology [17]. A scoping review was chosen to chart evidence relevant to interventions that enhance functional abilities in persons aged 50 years and over, and to identify knowledge gaps. This paper focuses on a sub-analysis of the results of an overarching scoping review conducted on the topic of age-friendly interventions and healthy ageing [18], aimed at examining evidence-based interventions that have been developed to create age-friendly communities. For the purpose of this review, we used the definition from the Food and Agriculture Organization of the United Nations [19] in classifying rural and remote areas as regions with smaller, dispersed populations and far away from main centres of services and facilities. Age-friendly communities, where environments and places support the building of intrinsic capacity and foster older people in maintaining and developing functional abilities, are critical to promoting healthy ageing. In the main scoping review, we found several interventions related to health and wellbeing that had been implemented in rural and remote areas, which involved older adults in activities that were important to them [20]. However, no published reviews have been identified on interventions to improve functional abilities. Hence, the emphasis of this review is on interventions focusing on the five domains of functional ability.

### Search

An overall project literature search was carried out on the following databases and platforms: CINAHL, Scopus, ProQuest Central, PubMed, EBSCOHost, APA PsycInfo, Carin.info, and the European Network for Rural Development Policy Projects database. Gray literature sources included government reports on national ageing strategies, the websites of national health departments, policy papers relevant to healthy ageing, and online newsletters of community organizations advocating healthy ageing initiatives. Additional records were also identified through a bibliographic hand search of included studies and relevant gray literature. Included in the search strategy were the following subject headings and key words: age-friendly, older people/persons, elder-friendly, rural ageing, rural gerontology, ageing in place, and later life (see the supplementary document). A search strategy was developed relating to ageing and healthy ageing (functional abilities) within rural and remote areas. Literature published from January 2010 up to March 9, 2021 were searched. This strategy was refined by a university librarian and through discussions with the team, which was comprised of experts in ageing and the WHO technical team. The search yielded a total of 9,130 citations. Data for this paper were generated based on following inclusion criteria:

### Inclusion criteria

- Published literature (academic and gray literature);
- Interventions and functional ability;
- Articles published in four international official languages (English, Chinese, French, and Spanish) between 2010 and March 2021;
- Literature that included older adults as defined by the articles, with a minimum age of 50 years; and.
- Any literature identified by the authors as involving the terms ‘rural’ or ‘remote’.

Excluded from the review were literature that focused on a non-community setting such as a nursing home or hospital setting, or studies that included findings from both rural/remote and urban areas but did not report findings by area for these two populations.

### Literature selection

**Fig. 1:**
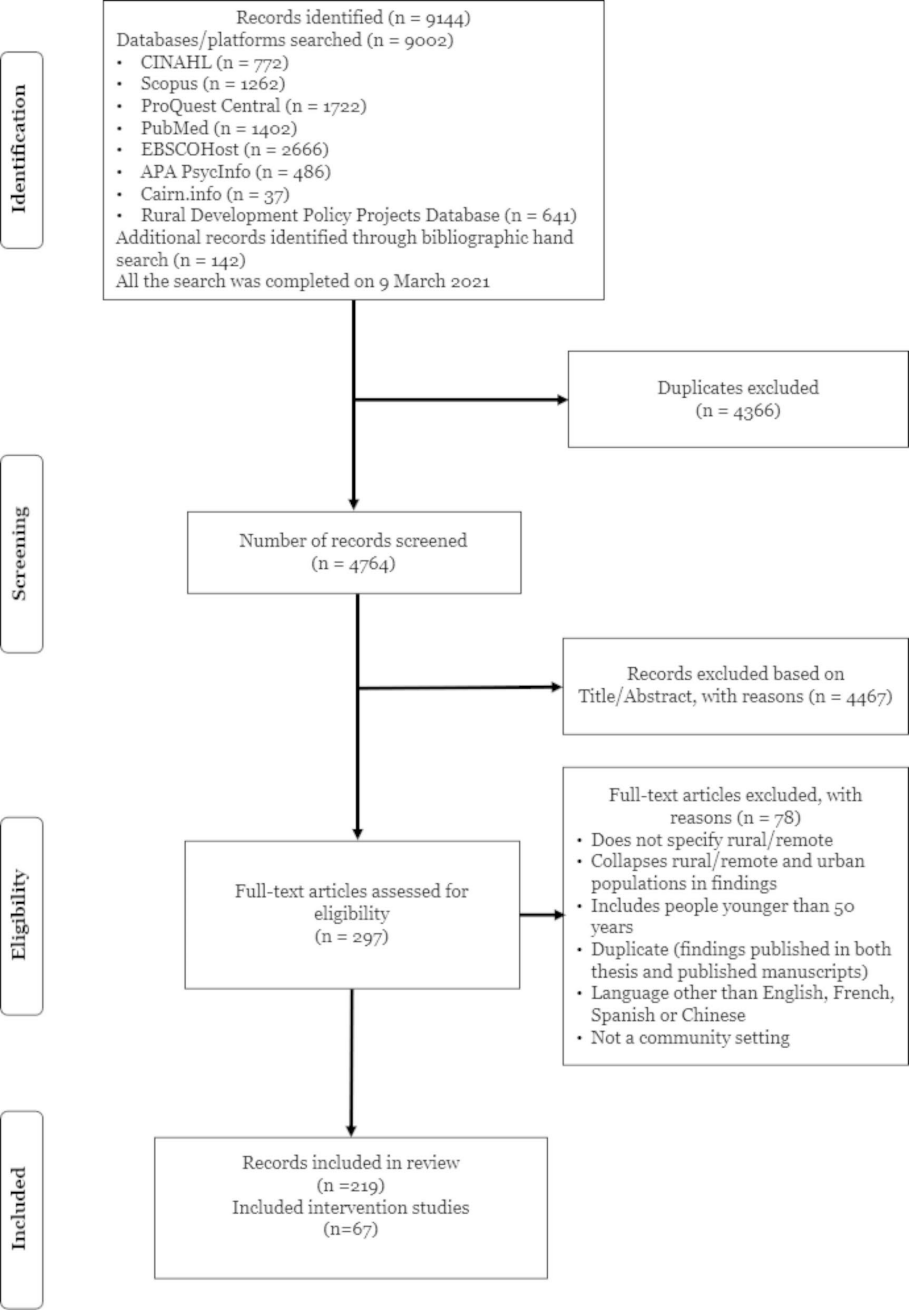
PRISMA-Scr extension for scoping reviews [21]

Literature selection was undertaken in two stages to enhance rigour. In the first stage, using Covidence (an online program used to screen papers retrieved in the research) three reviewers (JM, AK, IYZ) independently screened the titles and abstracts of all citations for potentially relevant articles by assessing each article against the pre-specified inclusion criteria. Any discrepancies were resolved with input from other authors (AL, AM, AO, CM, SN). In the second stage, the full-text article of each selected citation was assessed by at least two reviewers as in the earlier screening. The Preferred Reporting Items for Systematic Reviews and Meta-Analyses extension for Scoping Reviews (PRISMA-ScR) [21] and the PRISMA 2020 Statement [22] were utilized to show the results of the search and the process of by which articles were included in the study. Our database search unearthed 9002 articles and hand searching led to the identification of an additional 142 articles, for a total of 9144. After duplicates were removed, 4764 articles were included for title and abstract screening. We screened the full texts of 297 articles, and 67 articles (Supplementary document) were identified for data extraction. For detailed information, please refer to the PRISMA diagram flow chart in Fig. 1.

### Data extraction and synthesis

Scoping reviews do not involve appraising the quality of studies. However, a robust process was undertaken to identify the relevance of the papers retrieved to the aims of the review, according to the inclusion criteria. An Excel spreadsheet was used to extract data from the selected literature using the following headings: author/year; type of article; source; country; programme/intervention; methodology; concept; context; justification for rural and remote settings; and outcome measures. The focus and relevance of interventions in relation to the five domains of functional ability were also identified and verified among all seven reviewers, strictly following the description for each domain. Two of the included studies were selected and reviewed by the team to examine agreement across reviewers. Kendall’s coefficient of concordance (W) was used to examine the agreement of the assessments within the review team. The overall agreement among the seven reviewers was good, with W = 0.69 (p < 0.05).

## Functional ability domains

The five domains of functional ability were used to organize the interventions extracted in the analysis of evidence in this review. The categorizations and mapping were based on team discussions. The five domains are distinct but inter-related. Table 1 gives a description of all of these domains.

**Table 1:** Description of the five domains of functional ability

Functional Ability Domains	Description - World Report on Ageing and Health [8] (pp. 160–188)
Meet basic needs	Involves being able to afford to have an adequate diet, clothing, suitable housing, and healthcare and long-term care services. It also extends to having support to minimize the impact of economic shocks that may come with illness, disability, losing a spouse, or the means of making a livelihood.
Learn, grow, and make decisions	The ability to learn, grow, and make decisions includes efforts to continue to learn and apply knowledge, engage in problem solving, continue on a path to personal development, and be able to make choices.
Be mobile	Involves movement in all its forms, whether powered by the body (with or without an assistive device) or a vehicle. Mobility includes getting up from a chair or moving from a bed to a chair, walking for leisure, exercising, completing daily tasks, driving a car, and using public transport.
Build and maintain relationships	A broad range of relationships are important to older people, including their relationships with children and other family members, intimate relationships, and informal social relationships with friends, neighbours, colleagues, and acquaintances, as well as more formal relationships with community-service providers.
Contribute to society	Covers myriad contributions that older people make to their families and communities, such as assisting friends and neighbours, mentoring peers and younger people, and caring for family members and the wider community.

## Results

### Study characteristics

A total of 67 studies were identified, the designs of which included quasi-experimental studies (n = 44), randomized controlled trials (n = 22), and a descriptive study (with a naturally occurring intervention). Please refer to the supplementary document for detailed information on the included studies. Fifty studies evaluated outcomes and 17 evaluated processes. Of the 67 studies that were included, 59 were quantitative studies and 8 were mixed-method studies. All of the selected articles had been published between 2012 and 2021. Study groupings in the papers selected varied from one participant group to four groups. Sample sizes ranged from 2 to 4023 participants, and older people living in rural and remote communities were the primary focus of all of the studies. Forty-three studies reported gender ratios and ages (ranging from 50 to 82 years old).

The interventions were conducted across all six WHO Regions, with most found in the Americas (n = 29), the Western Pacific (n = 22), Europe (n = 5), and the South-East Asia region (n = 9). One study each was reported for the African region and the Eastern Mediterranean region. The studies were carried out in 18 countries, with the majority conducted in the United States, followed by Japan, South Korea, and Canada. Only 15 intervention studies were undertaken in middle- and low-income countries (see the Summary Table in the Supplementary document).

The interventions in this scoping review were all found to be healthcare-related, implemented in rural and remote settings with older people as participants, and to have outcomes aimed at improving health and wellbeing. Nine studies defined ‘rural and remote areas’ in reference to census/federal guidelines or characterized these areas in the study context as areas with a small number of inhabitants with limited resources, who did not have insurance coverage, and were of low economic status. Most studies raised issues and difficulties encountered by older people in rural and remote areas. The main issues identified from the data were high rates of chronic diseases [23–25] and inadequate access to health care and social services [26–30]. These were linked to challenging characteristics of rural and remote communities, such as low health literacy [31], limited transportation [32], poverty [33], and unsafe neighbourhoods or environments [23].These all negatively impact the functional ability of older adults in relation to meeting basic needs, being mobile, and building and maintaining relationships. Low health literary also prevented older adults from obtaining, processing, and understanding the basic health information needed to make appropriate health decisions.

### Types of interventions undertaken by older people in rural and remote settings

Five main types of interventions were conducted in rural and remote areas with older people: Community Services, Education and Training, Exercise and Physical Activity, Health Promotion Programmes, and Telehealth (Table 2). Health Promotion Programmes (n = 28, 41.8%) were the most frequently reported interventions in rural and remote areas. They included activities that ranged from health service delivery (mostly disease prevention) to practice guidelines on how to care for specific diseases and conditions (i.e., diabetes, anxiety). Some examples were home-delivered cognitive behavioural therapy for relieving anxiety [34], an mHealth-enabled integrated care model for patients with complex chronic conditions [35], and village-based interventions for depression [28]. These studies mainly reported positive changes in relation to the lifestyle, quality of life, and mental health of older adults as intervention outcomes.

The second most common type of intervention was Exercise and Physical Activity interventions (n = 16, 23.9%). These interventions such as a wearable Fitbit device [36] and an internet-based group exercise programme [37] were mainly designed for improving mobility. One study examined whether a multicomponent exercise programme based on independent home-training could enhance the cognitive function of older people [32]. There were two studies focusing on disease-specific symptoms related to physical functions [26, 38]. Most studies (n = 13) reported significant improvements in physical functioning.

Education sessions and modules were developed and delivered as Education and Training interventions (n = 14, 20.9%) aimed at improving the participants’ knowledge of common diseases and treatments, and changing the health behaviours of groups or individuals with specific health conditions. These interventions enabled older people to manage their own medications at home [39], improve their knowledge of diseases and conditions, and choose treatment options from the services available within their communities [25].

Telehealth interventions (n = 8, 11.9%) were similar to Education and Training interventions, as these were designed to deliver health education and training programmes through remote technological approaches, such as smart phones or teleconferencing software. Studies reported that older people’s ability to search for health information had improved [40] and that there was a significant reduction in the participants’ worries, anxiety, and depressive symptoms [41].

One study reported on a Community Service intervention in which a non-contributory pension programme was found to have positive effects on the nutritional intake (proteins and carbohydrates) of older people in rural areas [33].

**Table 2:** Types of intervention studies in rural and remote areas

Types of Interventions	Published Literature
Community Services (Pension)	1 (1.5%)
Education and Training	14 (20.9%)
Exercise and Physical Activity	16 (23.9%)
Health Promotion Programmes	28 (41.8%)
Telehealth	8 (11.9%)
**Total**	67 (100%)

### Relevance of studies and interventions to the WHO’s five domains of functional ability

Table 3 presents the distribution of the included studies across the WHO’s five domains of functional ability [9]. The categorizations of interventions under the five domains were finalized when all of the reviewers came to a consensus with reference to the WHO’s description of functional abilities (Table 1). Some interventions could be categorized under more than one domain of functional ability. All of the included studies were related to the ability to meet basic needs. About half of the studies were linked to the ability to learn, grow, and make decisions; and 40% were relevant to the ability to be mobile. Studies related to the ability to build and maintain relationships ‘with children and family, intimate partners, neighbours, and others’ made up 10% of the included studies [8], while those on the ability to contribute ‘by assisting friends, mentoring younger people, caring for family members, volunteering, pursuing cultural activities and working’ [8] made up 6%.

**Table 3:** Distribution of the included interventions by functional ability domains

Functional Ability Domains	No. of studies (*n* = 67)	Percentage
FA: Meet basic needs	67	100%
FA: Learn, grow, and make decisions	35	52%
FA: Be mobile	27	40%
FA: Build and maintain relationships	7	10%
FA: Contribute to society	4	6%

*FA: Functional Ability. *Some interventions cover more than one domain of functional ability

In terms of type, interventions were categorized as follows: Health Promotion Programmes (n = 28), Exercise and Physical Activity (n = 16), Education and Training (n = 14), Telehealth (n = 8), and Community Services (n = 1) (Table 4). The types of interventions were then mapped to the functional ability domains (Table 3). In the domain of meeting basic needs, interventions were related to Exercise and Physical Activity. In the domain of older people’s ability to learn, grow, and make decisions, most interventions involved Education and Training. Most of the outcomes that were measured (including access to healthcare, increased health knowledge and skills, improved physical function, decreased risk of falls, and the ability to manage their own health) were relevant to the domain of older people’s ability to meet their basic needs. Only one study reported that a real-time internet-based group exercise programme fostered the ability of older people to maintain and build relationships with other participants [37].

Across all intervention types, the functional ability domains that were least addressed were: (1) building and maintaining relationships; and (2) the ability to contribute to their families and communities. One of the few examples related to maintaining relationships and the ability to contribute was a Taiwanese study on older people living with chronic conditions. In that study, a community approach was utilized in which older residents were recruited as volunteers to coach other older people in medication safety [42]. No study was relevant to the functional ability domain of contributing to society.

**Table 4:** Distribution of functional ability (FA) domains by interventions

Types of Interventions	FA: Meet Basic Needs	FA: Learn, grow, and make decisions	FA: Be mobile	FA: Build and maintain relationships	FA: Contribute to society	Total FA Domains
Health Promotion Programmes	28	13	7	5	3	56
Exercise and Physical Activity	16	5	16	1	0	38
Education and Training	14	11	3	0	0	28
Telehealth	8	6	1	0	0	15
Community Services	1	0	0	0	0	1
**Total studies**	67*	35	27*	6*	3	

FA: Functional Ability. *Some interventions cover more than one domain of functional ability

## Discussion

The findings of this scoping review suggest that intervention studies involving older people in rural and remote areas have been limited to health-related and healthcare services. Our findings indicate that interventions for enhancing functional ability have primarily focused on increasing the ability of older people to meet their basic needs (such as increasing their physical and cognitive functions, supporting their mobility, and providing disease-specific healthcare access in rural and remote settings). Using the WHO’s five domains of functional ability to categorize interventions, the domains of building and maintaining relationships and contributing to communities were rarely addressed as outcomes of the intervention studies that were reviewed. The findings from this review further understanding of the service and resource gaps in addressing the functional trajectories, vulnerabilities, and inequities of older adults in rural and remote areas.

Most of the interventions in this review focused on health and physical activity, resulting in the predominance of one or two functional ability domains that were health specific, taking physical health measures as the key outcomes (such as gait speed, getting up from a chair, and grip strength). In these interventions, older peoples’ interactions with their environment in rural and remote settings have not been given full attention [9]. Not many interventions have been created to enhance these social interactions. This phenomenon is not limited to rural and remote settings, but has also been observed in urban areas [11]. The intrinsic capacity of individuals, the relevant environmental characteristics, and the interactions between them are central for older adults, although the interconnections among them are implicit [9]. Although the *Decade of Healthy Ageing; Plan of Action* calls on communities to foster the abilities of older people [43], limited efforts have been made to develop interventions to strengthen the ability of older adults to build and maintain relationships and contribute to their families and communities. Older people in rural communities have indicated a need for planning and for maintaining their independence in the face of changing and challenging environments. This requires a whole-of-society approach to ensure that policies and environments address the needs of older people in a more comprehensive way [44]. Governments need to consider providing health services that are both appropriate and accessible to older adults.

The review process unpacked the characteristics of some interventions, which were simultaneously compared to the functional ability domains as identified by the intervention outcomes. The outcomes of these interventions demonstrated improvements in some domains of functional ability, particularly those related to meeting basic needs and mobility. It has been documented in many other studies that interventions that enhanced some domains of the functional ability of older people tended to be indirectly related to the social participation [45, 46], civic engagement [47, 48], and inclusion [49, 50] aspects of the WHO age-friendly framework. The interventions examined in this review that evaluated outcomes did not account for the impact of external factors, such as a lack of infrastructure and resources, community support, and the safety of living environments and neighbourhoods in rural and remote settings. Outcomes such as physical function can be influenced by multiple socio-ecological factors [51, 52]. For example, in this review, exercise interventions that measured improvements in physical function in terms of gait, number of steps, and strength of the extremities, did not measure socio-ecological factors such as the ability of the participants to connect with co-participants or to socially participate while interventions are implemented [32]. A core action call of the WHO [8] for global change is about fostering more inclusive environments for older people to reduce ageism and inequities, as healthy ageing can only be achieved within supportive environments. Researchers, service providers, and policy makers who wish to target their efforts to inform the UN Decade of Healthy Ageing, should consider including all five domains of functional ability in their future strategies and interventions.

## Limitations

This review was based on a sub-analysis of data from an overarching scoping review [18]. There are some limitations to this review that need to be considered. The first is the paucity of studies that explicitly mentioned the use of the WHO functional ability framework to design the intervention. Although we included a broad range of terms as keywords that might have been relevant to functional abilities, such as physical activity, physical function, depressive symptoms and others, there might have been search limitations due to the different terminologies used in the included studies to describe healthy ageing or functional abilities. Second, gray literature identified in the literature search were excluded from this review, as they described initiatives for creating age-friendly communities that had not been evaluated. While analysing data in this review, we considered the heterogeneity of the study outcomes and the measures that were used, and decided that a more appropriate way to present evidence at this stage was to scope and map the available published intervention studies on rural and remote settings.

## Conclusion

Both the Decade of Healthy Ageing and the World Report on Ageing and Health [8, 43] emphasized the need to enhance older peoples’ intrinsic capacity across five functional ability domains. The framework of the WHO ICG was developed to ensure that integrated care and primary health services are accessible by any ageing group, including ageing populations in rural areas. People residing in rural and remote communities should have an equal opportunity to age in an environment that fosters ongoing lifelong learning and the ability to contribute to their communities while retaining their autonomy and health [13, 43]. However, existing studies on functional ability or intrinsic capacity in the context of healthy ageing lack consistency in the measurement of the processes or tools used to assess domains of intrinsic capacity among researchers and service providers. Relevant studies conducted in rural and remote areas offer limited insights. A standard measurement of intrinsic capacity for clinical or community settings has yet to be operationalized. Further validation of the concept of intrinsic capacity and standardized measurements of intrinsic capacity in the five domains are needed.

## Electronic supplementary material

Below is the link to the electronic supplementary material.

Supplementary Material 1

## Data Availability

The search strategies used in this scoping review are available in the supplementary document. All data used in this scoping review are from previously reported studies and datasets, which have been cited.
