# Supplementary material for: Interventions addressing functional abilities of older people in rural and remote areas: a scoping review of available evidence based on WHO functional ability domains
Source: BMC Geriatr. 2022 Oct 28;22:827. doi: 10.1186/s12877-022-03460-2 (PMC9615260; doi:10.1186/s12877-022-03460-2)
Supplement: Supplementary file 1 — Supplementary Material 1 [file 12877_2022_3460_MOESM1_ESM.docx]

**Supplementary Material**

***Supplementary File 1***

**Searching Terms and Results**

| **Database**  **Date** | **Search String** | **Results** |
| --- | --- | --- |
| CINAHL | **S1**  "age-friend*" OR "age-friendly program*" OR "age-friendly initiative*" OR "age-friendly environment*" OR "older people*" OR "older person*" OR "older adult*" OR "elder-friend*" OR "elder*" OR "50 years old and over" OR "60 years old and over" OR "rural ageing" OR "rural aging" OR "rural gerontology" OR "environmental gerontology" OR "liveable communit*" OR "soon to be old" OR "ageing in place" OR "later life" or “senior friendly” or “older population”  **S2**  (MM "Aging") OR (MM "Healthy Aging")  **S3**  (MM "Rural Population") OR (MM "Rural Health Services")  **S4**  rural or remote  **S5**  Program* or initiative or intervention or project or WHO domain or evaluat* OR checklist  **S6**  transport* OR access* OR infrastructure OR hous* OR home OR residence OR dwelling OR “social participation” OR “social involvement” OR “social engage*” OR “social active*” OR “social inclusion” OR respect OR “civic participation” OR “community participation” OR “community activ*” OR employ* OR job OR work OR communic* OR inform* OR “community support” OR “health service” OR “medical service” OR “outdoor space*” OR building OR recreation* OR “social determinants”  **S7** S1 OR S2  **S8** S3 OR S4  **S9** S5 AND S6  **S10** S7 AND S8 AND S9 | 772 |
| PubMed | (((Program*[Title/Abstract] OR initiative[Title/Abstract] OR intervention[Title/Abstract] OR project[Title/Abstract] OR WHO domain[Title/Abstract] OR evaluat*[Title/Abstract] OR checklist[Title/Abstract]) AND (transport* [Title/Abstract] OR access* [Title/Abstract] OR infrastructure [Title/Abstract] OR hous* [Title/Abstract] OR home [Title/Abstract] OR residence [Title/Abstract] OR dwelling [Title/Abstract] OR "social participation" [Title/Abstract] OR "social involvement" [Title/Abstract] OR "social engage*" [Title/Abstract] OR "social active*" [Title/Abstract] OR "social inclusion" [Title/Abstract] OR respect [Title/Abstract] OR "civic participation" [Title/Abstract] OR "community participation" [Title/Abstract] OR "community activ*" [Title/Abstract] OR employ* [Title/Abstract] OR job [Title/Abstract] OR work [Title/Abstract] OR communic* [Title/Abstract] OR inform* [Title/Abstract] OR "community support" [Title/Abstract] OR "health service" [Title/Abstract] OR "medical service" [Title/Abstract] OR "outdoor space*" [Title/Abstract] OR building [Title/Abstract] OR recreation* [Title/Abstract] OR "social determinants"[Title/Abstract])) AND (((((rural population[MeSH Terms]) OR (rural health services[MeSH Terms])) OR (rural health[MeSH Terms]))) OR (rural[Title/Abstract] OR remote[Title/Abstract]))) AND (((healthy aging[MeSH Terms]) OR (aging[MeSH Terms])) OR ("age-friend*"[Title/Abstract] OR "age-friendly program*"[Title/Abstract] OR "age-friendly initiative*"[Title/Abstract] OR "age-friendly environment*"[Title/Abstract] OR "older people*"[Title/Abstract] OR "older person*"[Title/Abstract] OR "older adult*"[Title/Abstract] OR "elder-friend*"[Title/Abstract] OR "elder*"[Title/Abstract] OR "50 years old[Title/Abstract] AND over"[Title/Abstract] OR "60 years old[Title/Abstract] AND over"[Title/Abstract] OR "rural ageing"[Title/Abstract] OR "rural aging"[Title/Abstract] OR "rural gerontology"[Title/Abstract] OR "environmental gerontology"[Title/Abstract] OR "liveable communit*"[Title/Abstract] OR "soon to be old"[Title/Abstract] OR "ageing in place"[Title/Abstract] OR "later life"[Title/Abstract] OR "senior friendly"[Title/Abstract] OR "older population"[Title/Abstract]))  Limits applied | 1402 |
| EBSCOHost  9/03/2021 | ("age-friend*" OR "age-friendly program*" OR "age-friendly initiative*" OR "age-friendly environment*" OR "older people*" OR "older person*" OR "older adult*" OR "elder-friend*" OR "elder*" OR "50 years old and over" OR "60 years old and over" OR "rural ageing" OR "rural aging" OR "rural gerontology" OR "environmental gerontology" OR "liveable communit*" OR "soon to be old" OR "ageing in place" OR "later life" OR "senior friendly" OR "older population") AND (rural OR remote) AND ((Program* OR initiative OR intervention OR project OR WHO domain OR evaluat* OR checklist)) AND ((transport* OR access* OR infrastructure OR hous* OR home OR residence OR dwelling OR "social participation" OR "social involvement" OR "social engage*" OR "social active*" OR "social inclusion" OR respect OR "civic participation" OR "community participation" OR "community activ*" OR employ* OR job OR work OR communic* OR inform* OR "community support" OR "health service" OR "medical service" OR "outdoor space*" OR building OR recreation* OR "social determinants"))  Search abstracts only  2010-present  Languages specified | 3478 (after limits applied)  Note that **2666** were exported since EBSCOHost automatically removes duplicates from exports |
| ProQuest | ab("age-friend*" OR "age-friendly program*" OR "age-friendly initiative*" OR "age-friendly environment*" OR "older people*" OR "older person*" OR "older adult*" OR "elder-friend*" OR "elder*" OR "50 years old and over" OR "60 years old and over" OR "rural ageing" OR "rural aging" OR "rural gerontology" OR "environmental gerontology" OR "liveable communit*" OR "soon to be old" OR "ageing in place" OR "later life" OR "senior friendly" OR "older population") AND ab(rural OR remote) AND ab(Program* OR initiative OR intervention OR project OR WHO domain OR evaluat* OR checklist) AND ab(transport* OR access* OR infrastructure OR hous* OR home OR residence OR dwelling OR "social participation" OR "social involvement" OR "social engage*" OR "social active*" OR "social inclusion" OR respect OR "civic participation" OR "community participation" OR "community activ*" OR employ* OR job OR work OR communic* OR inform* OR "community support" OR "health service" OR "medical service" OR "outdoor space*" OR building OR recreation* OR "social determinants") | 1722 |
| Scopus | ( TITLE-ABS-KEY ( "age-friend*" OR "age-friendly program*" OR "age-friendly initiative*" OR "age-friendly environment*" OR "older people*" OR "older person*" OR "older adult*" OR "elder-friend*" OR "elder*" OR "50 years old and over" OR "60 years old and over" OR "rural ageing" OR "rural aging" OR "rural gerontology" OR "environmental gerontology" OR "liveable communit*" OR "soon to be old" OR "ageing in place" OR "later life" OR "senior friendly" OR "older population" ) AND TITLE-ABS-KEY ( rural OR remote ) AND TITLE-ABS-KEY ( program* OR initiative OR intervention OR project OR who AND domain OR evaluat* OR checklist ) AND TITLE-ABS-KEY ( transport* OR access* OR infrastructure OR hous* OR home OR residence OR dwelling OR "social participation" OR "social involvement" OR "social engage*" OR "social active*" OR "social inclusion" OR respect OR "civic participation" OR "community participation" OR "community activ*" OR employ* OR job OR work OR communic* OR inform* OR "community support" OR "health service" OR "medical service" OR "outdoor space*" OR building OR recreation* OR "social determinants" ) ) AND PUBYEAR > 2009 AND ( LIMIT-TO ( LANGUAGE , "English" ) OR LIMIT-TO ( LANGUAGE , "Chinese" ) OR LIMIT-TO ( LANGUAGE , "Spanish" ) OR LIMIT-TO ( LANGUAGE , "Portuguese" ) OR LIMIT-TO ( LANGUAGE , "French" ) ) | 1262 |
| APA PsycInfo | ( "age-friend*" OR "age-friendly program*" OR "age-friendly initiative*" OR "age-friendly environment*" OR "older people*" OR "older person*" OR "older adult*" OR "elder-friend*" OR "elder*" OR "50 years old and over" OR "60 years old and over" OR "rural ageing" OR "rural aging" OR "rural gerontology" OR "environmental gerontology" OR "liveable communit*" OR "soon to be old" OR "ageing in place" OR "later life" or “senior friendly” or “older population” or (MM "Aging") OR (MM "Healthy Aging") ) AND AB ( (MM "Rural Population") OR (MM "Rural Health Services") or rural or remote ) AND AB ( Program* or initiative or intervention or project or WHO domain or evaluat* OR checklist ) AND AB ( transport* OR access* OR infrastructure OR hous* OR home OR residence OR dwelling OR “social participation” OR “social involvement” OR “social engage*” OR “social active*” OR “social inclusion” OR respect OR “civic participation” OR “community participation” OR “community activ*” OR employ* OR job OR work OR communic* OR inform* OR “community support” OR “health service” OR “medical service” OR “outdoor space*” OR building OR recreation* OR “social determinants” )  Limiters:  Abstracts only  2010-present  Languages specified: English, French, Chinese, Spanish | 486 |
| Cairn.info | («adapté à l’âge (des aînés)» OU «adaptés à l’âge (des aînés)» OU «qui réponde aux besoin des aînés» OU «qui répondent aux besoin des aînés» OU «favorable aux aînés» OU «favorables aux aînés» OU «adapté aux personnes âgées» OU «adaptés aux personnes âgées» OU «pour aînés» OU «programmes qui répondent aux besoin des aînés» OU « programmes favorables aux aînés» OU « programmes adaptés à l’âge (des aînés)» OU «initiatives qui répondent aux besoin des aînés» OU « initiatives favorables aux aînés» OU «initiatives adaptés à l’âge (des aînés)» OU «ville amie des aînés» OU «villes amies des aînés» OU «villes adaptées aux personnes âgées» OU «villes adaptées aux aînés» OU «aînés» OU «personnes âgées» OU «population vieillissante» OU «de 60 ans et plus» OU vieillissement dans les zones rurales» OU «vieillissement dans les régions rurales» OU «vieillissement dans les espaces ruraux» OU «vieillir en milieu rural» OU «gérontologie rurale» OU «vieillir sur place» OU «vieillir en place» OU «vieillissement chez soi» OU «vieillissement en place» OU «vieillissement sur place» OU «vieillir dans leurs logements»)  ET («zones rurales» OU «régions rurales» OU «espaces ruraux« OU «milieux ruraux» OU «régions éloignées» OU «régions reculées» OU «régions isolées» OU «zones éloignées» OU «zones reculées» OU «zones isolées») | 46 |
| Back search | Articles by the following authors were retrieved from PubMed (identified experts in age-friendly communities):   - Jeni Warburton - Rachel Winterton - Graham Rowles - Kieran Walsh - Mark Skinner - Vanessa Burholt - Thomas Scharf - Verena Menec | 142 |

***Supplementary File 2***

**Literature Functional Ability Summary Table**

|  | **Authors & Year** | **Type** | **Country** | **Study Aims** | **Study Design** | **Justification for setting** | **Intervention** | **Typology of Intervention** | **Outcomes** | **Type of Evaluation** |
| --- | --- | --- | --- | --- | --- | --- | --- | --- | --- | --- |
| 1 | Arnold et al. (2016) | Journal Article | United States | Evaluate if Fecal Occult Blood Test rates could be sustained over 3 years in rural US | Randomized controlled trial | In previous screening programs, fecal occult blood test screening is low in individuals from rural areas | Enhanced care; educational sessions; nurse support (combination) | Education and Training | FOBT screening rates were not sustained with any of the three interventions | Outcome |
| 2 | Batsis et al. (2021a) | Journal Article | United States | Evaluate the feasibility, acceptability, and effectiveness of integrating a wearable Fitbit device into a high-touch, multicomponent weight loss intervention at a local community aging center | Quasi-experimental (Pilot feasibility) | The program serves a predominantly rural catchment area where 14.5% of population were >65 years (compared to 11% in national average) | Individualized physical and nutrition program with use of Fitbit | Exercise and Physical Activity | Multicomponent obesity intervention (using a wearable device) was feasible and acceptable to older adults with obesity; significant improvement observed across physical function, late life function and subjective physical and mental scores | Outcomes & process |
| 3 | Batsis et al. (2021b) | Journal Article | United States | Determine the feasibility, acceptability and preliminary outcomes of an integrated technology-based health promotion intervention in rural-living, older adults using remote monitoring and synchronous video-based technology | Quasi-experimental | 78% of veterans in Maine lived in rural areas, and 2.1% in highly rural areas | An integrated technology-based health promotion intervention | Telehealth | Feasible and acceptable to older adults with obesity; significant improvements in weight loss and physical function | Outcomes & process |
| 4 | Blocker (2019)* | Dissertation/Thesis | United States | Increase physical activity among middle-aged and older adults living in rural Kansas, increase Alzheimer's disease knowledge through risk reduction education, and determine the unique barriers to physical activity and exercise in this rural Kansas cohort | Quasi-experimental | Insufficient accessibility of healthcare resources for older adults who live in rural areas | 10-week community-based education and exercise intervention program | Exercise and Physical Activity | Healthy lifestyle outcomes significantly improved for education & exercise group; other outcomes were not significantly improved for the education & exercise group compared to control or education-only group | Outcomes |
| 5 | Brenes et al. (2015) | Journal Article | United States | Examine the effects of telephone-delivered cognitive behavioral therapy (CBT) compared with telephone-delivered nondirective supportive therapy (NST) in rural older adults with generalized anxiety disorder | Randomized clinical trial | Older adults in rural areas are likely to have lower health literacy, which can impact on health decisions and overall health | Telephone-delivered cognitive behavioral therapy consisted of as many as 11 sessions (9 were required) | Telehealth | Significantly greater decline in worry severity, generalized anxiety disorder symptoms and depressive symptoms among participants in the telephone-delivered cognitive behavioral therapy group | Outcome |
| 6 | Chang et al. (2020) | Journal Article | Taiwan | Investigate the influence of fitness trackers with different goal setting strategies for older adults on physical activity | Randomized controlled trial | No justification for rural setting | Individualized goal setting based on their individual physical activity level | Exercise and Physical Activity | Compared to the universal group, the individual group experienced immediate effects within a short period of two weeks. However, there was no significant difference between the two groups | Outcome |
| 7 | Chueh et al. (2012) | Conference abstract/proceeding | Taiwan | Evaluate the effects of auricular acupressure (AA) on sleep disturbance on elderly aboriginal women | Quasi-experimental | Older adults are physically inactive, and in rural areas; there is limited availability of physical activity programs | Auricular acupressure | Health Promotion Programs | Improved sleep disturbance | Outcome |
| 8 | Crandall et al. (2019) | Journal Article | United States | Evaluate the impact of a game-centered mobile app (Bingocize®) on older adults' knowledge, skill, and confidence for managing aspects of their healthcare | Randomized controlled trial | No justification for rural setting | Health education and an exercise component in a group setting with use of app | Education and Training | Increased skills, confidence and knowledge of health | Outcome |
| 9 | de Batlle et al. (2020) | Journal Article | Spain | Assess the acceptability, usability, and satisfaction of an mHealth-enabled integrated care model for complex chronic patients in both patients and health professionals | Quasi-experimental (Feasibility) | Many rural adults with diabetes have limited access to diabetes educators; telemedicine is a feasible and acceptable approach to providing services. | Wearable device and integrated care model | Health Promotion Programs | Patient acceptability and usability were high but staff acceptability and usability were low and average respectively, although actual use of technology was high | Outcomes & process |
| 10 | DiNapoli et al. (2017) | Journal Article | United States | Examine the effects of home-delivered cognitive-behavioral therapy (CBT) for depression on anxiety symptoms in an ethnically diverse, low resource, and medically frail sample of rural, older adults | Randomized controlled trial | Unmet treatment needs among rural adults | Cognitive behavioral therapy | Health Promotion Programs | Improved quality of life and reduction in psychological symptoms | Outcome |
| 11 | Dongre et al. (2012) | Journal Article | India | Evaluate the effect of a community-managed palliative care program on perceived quality of life in the elderly in the project villages in rural Tamil Nadu. | Randomized controlled trial | No justification for rural setting | Community-Managed Palliative Care Program | Health Promotion Programs | the model of “community-managed” palliative care program improved perceived physical quality of life and psychological support among the elderly; no impact on social relationship and environment domains | Outcome |
| 12 | Dumitrache et al. (2017) | Journal Article | Spain | Explore the effectiveness of an intervention program aiming at improving quality of life in a group of community-dwelling older adults living in a depopulated rural area in Orense, Galicia, Spain | Quasi-experimental (Pre-post) | Ageing has not been studied extensively, unbalanced distribution of resources among rural older adults compared to urban adults; Spanish ageing policies do not give specific recommendations about older people | Workshops aimed at improving cognitive and physical function | Health Promotion Programs | Intervention reduced risk of cognitive impairment; participants believed that opportunity for leisure activities increased which also corresponded with increased in perception of mental health | Outcome |
| 13 | Dye et al. (2018) | Journal Article | United States | Pilot test a model to reduce hospital readmissions and emergency department use of rural, older adults with chronic diseases discharged from home health services (HHS) through the use of volunteers | Quasi-experimental (Pilot feasibility) | Rural populations experience poorer health outcomes compared to urban counterparts due to lower socioeconomic status, reduced access to health services, and incidence of chronic disease; rural adults are frequently readmitted for the same health conditions | Use of volunteer community members who were trained as health coaches | Health Promotion Programs | Program participants were able to monitor and track their chronic health conditions, make positive lifestyle changes, reduce incidence of falls, pneumonia and flu; No significant differences in ED/hospital admission rates | Outcome |
| 14 | Elder et al. (2016)* | Journal Article | United States | Examine satisfaction with and outcomes of a real-time Internet-based group exercise program for older adults | Quasi-experimental | Limited access to health care services in rural areas; physical activity can be partially dependent on access to safe, low-cost, inviting environments; limited opportunities for indoor physical activity | 10-week distance group exercise program | Exercise and Physical Activity | Intervention found to be valuable by participants; significant improvement in physical function | Outcomes & process |
| 15 | Ford et al. (2017) | Journal Article | United States | To evaluate the effectiveness of a quality improvement intervention to increase delivery of 2 evidence-based health promotion workshops, Stepping On and Chronic Disease Self-Management Program (CDSMP), in rural communities. | Cluster-randomized wait-list control group design | Higher proportion of older people in rural setting; difficult to engage older people in rural areas with health-promotion programs; inadequate financial or staff resources to implement these programs | Training and coaching county aging unit staff to provide workshops | Health Promotion Programs | Significant reduction in falls, decline in emergency department visits | Outcome |
| 16 | Fouladbakhsh et al. (2011) | Journal Article | United States | Evaluate the effects of an intervention to teach older adults in a rural community about the use of nondrug therapies for self-treatment of pain | Quasi-experimental | Limited access to medical care and financial resources mean that rural residents may prefer to opt for self-treatment in health care; higher prevalence of pain and non-drug therapies for management with less information/access about these therapies | Education | Education and Training | Significant increase in the use of non-drug treatments and a decrease in pain-related distress and pain scores | Outcome |
| 17 | Giesbrecht et al. (2015) | Journal Article | Canada | Determine the acceptability and feasibility of administering an mHealth wheelchair skills training program safely and effectively with two participants of different skill levels | Quasi-experimental (Pre-post feasibility) | Need for alternative and innovative electronic and mobile technology strategies to deliver health-related services in rural settings | Training and home education program | Education and Training | Program was acceptable and feasible, some initial issues with configuration of program | Outcomes & process |
| 18 | Haque et al. (2020) | Journal Article | Bangladesh | Pelvic floor and mobility exercises were shown to be effective in managing incontinence in a cluster‐randomized trial (CRT) of village women aged 60 to 75 years in Bangladesh. The present analysis examines continence 12 months after the CRT and exercise program implementation with village paramedics as preceptors. | Quasi-experimental (Pre-post) | Few resources at community clinics in rural area to meet needs | Group exercise program | Exercise and Physical Activity | Improvement in continence | Outcome |
| 19 | Hockin (2013) | Dissertation/Thesis | United States | Evaluate the relationship between cognitive training (CT) and problem solving in older adults 12 to 24 months post CT intervention | Quasi-experimental (Hybrid, ex post facto) | No justification for rural setting | Cognitive training | Health Promotion Programs | No significant relationships between cognitive training or demographic variables and problem solving were found | Outcome |
| 20 | Hosseini et al. (2013) | Journal Article | Iran | Define the effect of nurse home visits on self-care self-efficacy of the elderly in rural areas | Quasi-experimental (randomized, pre-post) | Proportionally, there are more older adults living in rural areas compared to urban areas; importance of health-promoting self-care behaviors in increasing quality of life in the elderly | Five home visit programs and one group session by a nurse during 6 weeks, and included two different sections of education and nursing interventions administered based on needs assessment and determination of the tasks for the clients and their families. | Health Promotion Programs | A significant difference was seen in the mean total scores of self-care self-efficacy and its subscales | Outcome |
| 21 | Hsu et al. (2018) | Journal Article | Taiwan | Implement and evaluate a cross-disciplinary intervention program using two approaches for community-based older adults in Taichung, Taiwan | Quasi-experimental | No justification for rural setting | Cross-disciplinary intervention program using personal-and-digital approaches aimed at promoting healthy ageing | Telehealth | Among rural population, the ability to search for health information improved, as did self-rated health. | Outcome |
| 22 | Hu et al. (2017) | Journal Article | China | Evaluate the effectiveness of a synthetic intervention model aimed at preventing type 2 diabetes and controlling plasma glucose, body weight and waist circumference in elderly individuals with prediabetes in rural China | Randomized controlled trial | Previous interventions for diabetes have been conducted in resource-intensive areas; however, these may not work in rural areas with social, economic and cultural differences | Lifestyle health promotion intervention for diabetes (synthetic intervention model consisted of lifestyle education, counselling, self-monitoring of blood glucose, and setting up a Health Each Other Group) | Health Promotion Programs | Intervention contributed to weight loss and decrease in fasting glucose | Outcome |
| 23 | Izquierdo et al. (2010) | Journal Article | United States | Examine the changes in waist circumference (WC) and body mass index (BMI) in older adults enrolled in a diabetes telemedicine program | Randomized controlled trial | Potential for telemedicine to deliver care to individuals in rural areas with diabetes who have poor access to health care | Diabetes telemedicine program | Telehealth | The program improved self-reported diet/exercise related knowledge and practices; changes were associated with improved physical measure (waist circumference and BMI) | Outcome |
| 24 | Jang et al. (2018a) | Journal Article | South Korea | Evaluate the effectiveness of a 6-month multicomponent intervention on physical function in socioeconomically vulnerable older adults in rural communities. The study also evaluated the effectiveness of the intervention on frailty and other geriatric syndromes, sustained benefit at 12 months, and baseline characteristics associated with poor response. | Quasi-experimental (Designed-delay pre-post) | Prevalence frailty especially high among older adults who live in rural areas, and since there are fewer facilities/resources, they're at higher risk of functional decline; effectiveness/feasibility of interventions addressing this haven't been explored that extensively | Multicomponent program for physical and mental wellbeing | Health Promotion Programs | Improved physical function, frailty, sarcopenia, depressive symptoms and nutritional status. | Outcome |
| 25 | Jang et al. (2018b) | Journal Article | South Korea | We aimed to evaluate whether a wearable device and mobile-based intermittent coaching or self-management could increase physical activity and health outcomes of small groups of older adults in rural areas | Quasi-experimental (Feasibility) | Older adults living in rural areas have limited availability for health care services and tend to have lower socioeconomic status, educational level, higher prevalence of living alone, multi-morbidity, frailty and disability | Wearable device and mobile-based coaching and self-management | Health Promotion Programs | The Smart Walk program improved physical fitness, anthropometric measurements and geriatric assessment | Outcome |
| 26 | Jeon et al. (2014) | Journal Article | South Korea | Devise a recurrent fall prevention program for elderly women in rural areas | Randomized controlled trial | Older people who live in rural communities have less access to treatment and education for their recovery of physical and psychological impairments caused by falling compared to urban adults | Physical activity and education - fall prevention program | Exercise and Physical Activity | Improves muscle strength, endurance, balance and psychological outcomes | Outcome |
| 27 | Jindo et al. (2016) | Journal Article | Japan | Compare the effect on LEPF (lower extremity physical function) between an exercise intervention with and without the use of a pedometer | Quasi-experimental | No justification for rural setting | Fall-prevention exercise | Exercise and Physical Activity | Some improvement in physical function, findings suggest that the use of a pedometer is useful to improve LEPF | Outcome |
| 28 | Jindo et al. (2017) | Journal Article | Japan | Investigate how daily life physical activity modulates the effects of an exercise program on LEPF (lower extremity physical function) | Quasi-experimental | No justification for rural setting | Fall-prevention program using exercise | Exercise and Physical Activity | Square-stepping exercise can improve lower extremity physical function | Outcome |
| 29 | Jo et al. (2016) | Journal Article | South Korea | Apply the “Happy Together” integrated cognitive behavior program for elderly people with dementia who are living at home | Quasi-experimental | Elderly people with dementia tend to be cared for at home in rural areas, with family typically being the main caregiver | Integrated cognitive behavioral training | Health Promotion Programs | Significant change with activities of daily life and burden for caregivers | Outcome |
| 30 | Kawamoto et al. (2015) | Journal Article | Japan | Determine whether baseline arterial stiffness is independently associated with improvement in functional abilities after exercise training | Quasi-experimental (Pre-post) | No justification for rural setting | 12-week Nordic walking program | Exercise and Physical Activity | Baseline brachial ankle stiffness may be a predictor of functional ability after exercise | Outcome |
| 31 | Kim et al. (2017) | Journal Article | South Korea | Evaluated the effectiveness of telemedicine in relation to cognitive changes in patients with dementia | Quasi-experimental (non-randomized controlled trial) | No justification for rural setting | Telemedicine services | Telehealth | Cognitive decline was significantly lower in the telemedicine group for the less severe initial cognitive performance subgroup than more severe cognitive performance subgroup | Outcome |
| 32 | Ko et al. (2021) | Journal Article | Myanmar | Measure the effect of door-to-door eye health education in improving knowledge and attitude toward cataracts and the use of eyecare services among the elderly residing in a rural area of Naypyitaw Union Territory, Myanmar | Quasi-experimental | High prevalence of eye diseases in identified rural town, with no recorded cataract intervention | Cataract education delivered at home | Education and Training | Significantly increased knowledge and attitude toward cataracts and the use of eyecare services among the elderly with cataracts. | Outcome |
| 33 | Köhler et al. (2014) | Journal Article | Germany | Evaluate an already existing regional dementia network | Randomized controlled trial | Rapidly aging population with limited local medical care | Regional dementia network | Health Promotion Programs | Interdisciplinary regional network provides significant advantages with getting medication and access to appropriate specialists; No significant difference in quality of life nor overall effects or treatment by time effects | Outcome |
| 34 | Krajnik (2018) | Dissertation/Thesis | United States | Investigate the feasibility of providing theory-based computer and Internet skills training to rural-dwelling older adults and explore the effects on their ability to locate and evaluate online health information | Quasi-experimental (Pre-post feasibility) | The computer and Internet health information concerns of the rural older adults have not been explored, even though they have limited access to healthcare providers and tend to experience poorer health | Educational program around computer and Internet health training | Education and Training | Found to be feasible and improved knowledge and self-perceptions of electronic health literacy | Outcomes & process |
| 35 | Kume et al. (2019) | Journal Article | Japan | Examine whether the multicomponent exercise program based on an independent home-training can become effective strategy for Japanese older people in a depopulated rural region | Quasi-experimental | Very high >65 population in rural city (44% in 2017), and the need for exercise among older adults especially due to limited transport access | Multicomponent exercise program | Exercise and Physical Activity | Exercise program based on home-training can be effective for enhancements of the gait ability and the executive function for older individuals in a depopulated rural region | Outcome |
| 36 | Lembeck et al. (2019) | Journal Article | Denmark | Determine whether discharge planning including a single follow-up home visit reduces readmission rate | Randomized controlled trial | Hospitalizations due to ambulatory care sensitive conditions are greater among people with certain sociodemographic characteristics (suggestive that rural may be a characteristic of interest) | Follow up home visit following hospital discharge (hospital at home intervention) | Health Promotion Programs | No effect on readmission rates nor on any secondary outcomes | Outcome |
| 37 | Lin et al. (2015) | Journal Article | Taiwan | Tests the effectiveness of a diet and exercise program on the MetS biomarkers in older community residents with metabolic syndrome | Quasi-experimental | Few studies have studied the effect of a combined diet and exercise program on managing metabolic syndrome (MetS) in individuals 65 years and older and living in Taiwan’s rural areas | Combined diet and exercise program | Health Promotion Programs | Intervention group had significantly improved anthropometric measurements and biomarkers | Outcome |
| 38 | MacIntyre et al. (2019) | Journal Article | Canada | Improve osteoporosis care and decrease bone fracture risk in a population of patients 65 years of age and older | Quasi-experimental | No justification for rural setting | Invitation to self-refer for osteoporosis and nurse-led intervention | Health Promotion Programs | Self-referral program resulted in an increase in the number of people who tested for bone mineral density; proportion of low risk participants recently tested higher than those previously tested for bone mineral density | Outcome |
| 39 | Mangalvedhe et al. (2017) | Journal Article | India | The present study was conducted to know the impact of Otago Exercise Intervention on risk of falls in the community in a village in Mysore district | Quasi-experimental (Pre-post) | No justification for rural setting | Otago exercise intervention | Exercise and Physical Activity | Exercise intervention effective in reducing risk of falls, improvement in physical function | Outcome |
| 40 | Matsubayashi et al. (2016) | Journal Article | Japan | This study examined whether low-frequency group exercise improved the motor functions of community-dwelling elderly people in a rural area when combined with home exercise with self-monitoring | Quasi-experimental | Reduced human resources in rural areas; issues with good transportation network in these areas so an intervention with few should be explored | Group exercise and home exercise | Exercise and Physical Activity | Intervention improved physical function | Outcome |
| 41 | McMahon et al. (2016) | Journal Article | United States | To assess the feasibility of a new intervention, Ready~Steady, in terms of demand, acceptability, implementation, and limited efficacy. | Randomized controlled trial | No justification for rural setting | Program providing social support and falls-prevention, inclusive of an app | Exercise and Physical Activity | Acceptable to population and good implementation fidelity. Improved physical function | Outcomes & process |
| 42 | Naik et al. (2012)* | Journal Article | United States | Test the acceptability, feasibility and preliminary outcomes of a telephone-delivered coaching intervention for rural-dwelling older adults with uncontrolled diabetes and comorbid, clinically significant depressive symptoms. | Quasi-experimental | Treatment options for diabetes and depression are rarely available for patients in rural settings | Telehealth & diabetes (disease-specific) | Telehealth | Participants responded positively to the collaborative goal setting and action-planning; clinically significant improvements observed to outcomes relating to diabetes and depression | Outcomes & process |
| 43 | Nott et al. (2019) | Journal Article | Australia | This pilot trial examined the feasibility and effectiveness of “Ageing Well,” a community‐based program for improving cognitive skills and mobility of rural older people. | Quasi-experimental (Non‐randomized, wait‐list–controlled pilot trial) | Geographic isolation; dispersed population and vulnerability to economic restructuring means that interventions for healthy aging need to take a multidimensional approach; lack of studies including older people living in regional/rural communities | Dual‐tasking activities to improve motor and cognitive abilities | Health Promotion Programs | Ageing Well program is feasible, safe and acceptable; data supports some effectiveness | Outcomes & process |
| 44 | Oh et al. (2020a) | Journal Article | South Korea | Examine the feasibility and effectiveness of a village-based intervention for depression targeting older adults living in rural areas | Randomized controlled trial | Limited access to treatment for late-life depression among older people living in rural areas | Case management and group-based activities for depression | Health Promotion Programs | Intervention did not reduce depressive symptoms in the older population overall, which was probably due to the inclusion of large numbers of healthy elderly individuals; intervention lowered the risk of progression to severe depression among at-risk older adults, and increased the social network more than five-fold | Outcome |
| 45 | Oh et al. (2020b) | Journal Article | South Korea | This study investigated the effectiveness of an integrated intervention program combining self-directed home-based resistance training with health education for older adults with knee osteoarthritis living in a rural area | Randomized controlled trial | Impact of integrated intervention programs among people with osteoarthritis in rural areas not known | Health education and home-based resistance training | Education and Training | Improvement in mobility/physical function | Outcome |
| 46 | Ohta et al. (2021)* | Journal Article | Japan | Study hypothesized that social cognitive theory (SCT)-based educational interventions for healthcare participation can improve the self-efficacy of older rural citizens in participating in their health management without any difficulties | Quasi-experimental | People in rural areas tend to have lower health literacy skills, social norms mean that people consider living without help virtue (stronger among older people) | Social cognitive theory (SCT)-based educational intervention | Education and Training | Social cognitive theory-based educational interventions can positively impact self-efficacy in healthcare participation | Outcome |
| 47 | Olakehinde et al. (2019) | Journal Article | Nigeria | Investigate the feasibility and clinical impact of a psychosocial intervention, Cognitive Stimulation Therapy (CST), to help manage dementia in a rural setting in Nigeria | Quasi-experimental (Pre-post feasibility) | No justification for a rural setting | Cognitive Stimulation Therapy (CST) | Health Promotion Programs | Cognitive Stimulation Therapy is feasible in this setting; Significant improvements in cognitive function, quality of life (physical, psychosocial and environmental domains), physical function, neuro-psychiatric symptoms and carer burden | Outcomes & process |
| 48 | Paschoa & Ashton (2016)* | Journal Article | United States | Evaluate a group exercise program for older adults living in a rural community | Quasi-experimental | Physical inactivity is higher among rural populations, this may be attributed to: 1) natural social networks of older adults may not reinforce an active lifestyle; 2) older adults fear exercising outside due to unsafe neighbourhoods or natural environments; 3) lack of knowledge of the importance of exercising to improve quality of life, health status, daily functioning, health care costs and knowledge on how to exercise safely | Exercise programs focused on reducing sedentary behavior; improved physical function and participants were satisfied with the program | Exercise and Physical Activity | Improved knowledge and physical function; participants were satisfied with the program | Outcome & process |
| 49 | Pesut et al. (2017)* | Journal Article | Canada | Pilot a nurse-led navigation service to provide early palliative support for rural older adults and their families living at home with advancing chronic illness | Quasi-experimental (pilot) | Few available services to support rural older people receiving palliative care, even though they require more transition compared to urban residents; rural communities also have the capacity to provide high quality care as there are both personal and professional relationships co-existing | Palliative care support delivered at home | Health Promotion Programs | Intervention is a promising intervention to meet the needs of population | Outcome & process |
| 50 | Rachasrimuang et al. (2018) | Journal Article | Thailand | Evaluate the effectiveness of home visits programme by a youth volunteer on the health-related quality of life and depression among elderly persons living in a rural community | Cluster randomized controlled trial | No justification for rural setting | Trained youth (Grades 6-9) volunteers to visit older people in their homes | Health Promotion Programs | Improved health and depression score | Outcome |
| 51 | Rana et al. (2010) | Journal Article | Bangladesh | To examine changes in self-reported arthritis-related illness and self-rated health as a result of a health education intervention, and the association between self-reported arthritis-related illness and self-rated health | Quasi-experimental | No justification for rural setting | Home-based physical activities, health advice | Exercise and Physical Activity | Those who were compliant to health advice were more likely to report improvement in arthritic-related illnesses; those who were literate and non-poor were more likely to report positive health | Outcome |
| 52 | Rizkalla (2015) | Dissertation/Thesis | Canada | 1. Develop a novel collaborative approach to improving access to cognitive training in rural settings; 2. Evaluate the efficacy of a multicomponent cognitive training program for improving cognition in normal elderly persons | Randomized controlled trial | Lack of access to specialty services for rural older adults in Canada | Three training modules to improve cognitive function | Education and Training | Improved executive function and memory but not psychosocial ability | Outcome |
| 53 | Sarkar et al. (2017) | Journal Article | India | Assess the impact of attendance at a community-based daycare center in rural Puducherry, India, on depression, cognitive impairment (CI) and quality of life of the elderly | Quasi-experimental (Pre-post) | Majority of elderly population live in rural areas; rural elderly were worst affected by changing family structure in Indian villages as well as migration of younger people to urban areas which makes them more vulnerable to access social services and health facilities. | Problem-solving therapy (psychological therapy) over 4-5 sessions | Health Promotion Programs | Attendance at daycare center reduced risk of depression by half; improved social domain of quality of life score | Outcome |
| 54 | Schweickert et al. (2011) | Journal Article | United States | Evaluate the effectiveness of delivering stroke education to elderly individuals through telehealth versus in person stroke prevention education methods | Quasi-experimental (Pre-post) | Stroke is prevalence among elderly peoples; however, education is difficult to deliver in rural/remote regions | Mixed telehealth and in-person education | Telehealth | Telehealth stroke education is feasible; there were no between-group differences in changes in knowledge or likelihood in making behavioral changes but changes were observed pre-post intervention for intervention group | Outcomes & process |
| 55 | Scogin et al. (2014) | Journal Article | United States | Examine the effects of home-delivered cognitive-behavior therapy (CBT) on depressive symptoms among rural, diverse, and vulnerable older adults | Randomized controlled trial | High number of older adults living in rural areas and risk of mental health in this group; paucity of research of cognitive behavioral therapy | Cognitive behavior therapy | Health Promotion Programs | Diverse range of adults responded to the intervention and was effective in lowering depression symptom severity scores | Outcome |
| 56 | Seangpraw et al. (2019) | Journal Article | Thailand | Evaluate the dietary behavior modification program Dietary Approaches to Stop Hypertension (DASH) with self-efficacy to reduce the risk of hypertension among the elderly as well as to motivate elderly people to have healthy dietary behaviors in order to prevent hypertension | Quasi-experimental (pre-post test) | No justification for rural setting | Dietary education group session, training on improved healthy lifestyle (diet, physical activity) | Education and Training | Improved perceived severity, self-efficacy and preventive behaviors; no actual changes in anthropometic measurements, physical activity and smoking levels | Outcome |
| 57 | Shreffler-Grant et al. (2018) | Journal Article | United States | The purpose is to describe a feasibility study of a skill-building intervention to enhance health literacy about complementary and alternative (CAM) therapies among older rural adults and share lessons learned. | Quasi-experimental (Pre-post feasibility) | The independent nature of rural dwellers, scarcity of rural health care resources, prevalence of chronic health conditions among older rural adults, a general lack of knowledge about complementary and alternative medicines (CAM), and concern with the quality of available information about CAM make it critical that older rural consumers have sufficient health literacy about CAM. | An intervention to enhance complementary and alternative therapies health literacy; modules were presented face to face and by webinar | Education and Training | Intervention was implemented and evaluation and the team are exploring implementing it to other communities | Process |
| 58 | Smith et al. (2017) | Journal Article | United States | Work with local rural organizations to develop an evidence-based hatha yoga program intended to improve core strength and balance to reduce falls risk. | Quasi-experimental (Single-arm pilot study) | No justification for rural setting | Yoga (classes and at home) | Exercise and Physical Activity | Intervention was acceptable | Process |
| 59 | Sowle (2015)* | Dissertation/Thesis | United States | Evaluate the Living (well through) Intergenerational Fitness and Exercise (LIFE) Program in rural Iowa counties | Quasi-experimental | Older rural adults have higher rates of chronic disease, are further from health/fitness resources, are less active but also prefer programs that are free, accessible, fun and social | Physical, social, emotional, intellectual and vocational program | Health Promotion Programs | Increased physical activity , improved self-efficacy, well-received | Outcomes & process |
| 60 | Takada et al. (2018) | Journal Article | Japan | To determine the effect of an oral self-care program on oral, cognitive, and daily performance functions in rural community-dwelling older people with mild cognitive impairment. | Quasi-experimental | Dementia is concerning especially with there are limited social/economic resources so preventive interventions are needed | Oral self-care program | Health Promotion Programs | Programme may be an effective means to delay oral, physical, and cognitive decline in rural, community-dwelling older people with mild cognitive impairment | Outcome |
| 61 | Téllez-Rojo et al. (2013) | Meeting abstract | Mexico | Examine the nutritional impact of a non-contributory pension programme among older people in rural areas to improve the welfare of the elderly | Quasi-experimental (regression discontinuity) | Elderly people in Mexico experience poverty with limited access to social protection systems, making this population vulnerable, particularly for those who live in rural areas | Pension | Community Service (Pension) | Significant effect on protein, carbohydrate intake. No impact on BMI | Outcome |
| 62 | Theeke et al. (2015)* | Journal Article | United States | Present the initial feasibility and acceptability of LISTEN (Loneliness Intervention using Story Theory to Enhance Nursing-sensitive outcomes), a new intervention for loneliness | Randomized controlled trial | No justification for rural setting | Loneliness Intervention using Story Theory to Enhance Nursing-sensitive outcomes, a new intervention for loneliness | Health Promotion Programs | LISTEN was evaluated as feasible to deliver with no attrition and near perfect attendance; Participants ranked LISTEN as highly acceptable for diminishing loneliness with participants requesting a continuation of the program or development of additional sessions | Process |
| 63 | Volandes et al. (2011) | Journal Article | United States | Evaluate the end-of-life preferences of elderly patients in rural communities and whether preferences are associated with level of health literacy. | Randomized controlled trial | No prior studies on end-of-life decision-making have assessed the effects of health literacy in rural populations or the use of a video decision aid to better inform rural patients | Verbal description follows by video decision aid to assist education/decision making around advanced dementia care | Education and Training | People with higher health literacy referred comfort care, as did people who viewed the video decision aid | Outcome |
| 64 | Walters et al. (2017) | Journal Article | Netherlands | Determine the short- and medium term effects of an intervention to support workers in providing preventive activities for older adults | Quasi-experimental | No justification for rural setting | Training program for preventive health problem-activities performed by home health care professionals | Education and Training | Did not significantly improve outcomes; however, there is potential that health promotion could be effective with enhanced delivery | Outcome |
| 65 | Wang et al. (2013) | Journal Article | Taiwan | To promote medication safety among rural elders with chronic illnesses | Randomized controlled trial | Challenge for rural health professionals to promote medication safety among older adults taking multiple medications | Volunteer coaching program | Education and Training | the volunteer coaching group improved their knowledge of medication safety; no change in attitude after the two-month study period; the group demonstrated three improved medication safety behaviors compared to the routine care group; volunteer coaching program and instructions with pictorial aids can provide a reference for community health professionals who wish to improve the medication safety of chronically ill elders | Outcome |
| 66 | West et al. (2010) | Journal Article | United States | Describe the use of telemedicine for setting goals for behavior change and examine the success in achieving these goals in rural underserved older adults with diabetes | Descriptive (based on another study which is an RCT) | Many rural adults with diabetes have limited access to diabetes educators; telemedicine is a feasible and acceptable approach to providing services. | Televisits with diabetes educator | Telehealth | Most behavioral goals were improved on or met | Outcome |
| 67 | Xu et al. (2020) | Journal Article | China | Investigate whether an integrated health care intervention improved health-related quality of life (HRQoL) and well-being among older patients with hypertension in rural China | Randomized controlled trial | Rural China's healthcare system is hospital-centered and fragmented, leading to poor continuity of care | integrated health care intervention | Health Promotion Programs | Decrease in reports of anxiety/depression; patients’ health awareness and knowledge improved; rate of compliance with a healthy lifestyle was low | Outcome |
